# Histological assessment of granulomas in natural and experimental *Schistosoma mansoni* infections using whole slide imaging

**DOI:** 10.1371/journal.pone.0184696

**Published:** 2017-09-13

**Authors:** Kátia B. Amaral, Thiago P. Silva, Felipe F. Dias, Kássia K. Malta, Florence M. Rosa, Sócrates F. Costa-Neto, Rosana Gentile, Rossana C. N. Melo

**Affiliations:** 1 Laboratory of Cellular Biology, Department of Biology, Federal University of Juiz de Fora (UFJF), Juiz de Fora, MG, Brazil; 2 Laboratory of Parasitology, Department of Parasitology, Microbiology and Immunology, Federal University of Juiz de Fora (UFJF), Juiz de Fora, MG, Brazil; 3 Laboratory of Biology and Parasitology of Wild Reservoir Mammals, Oswaldo Cruz Foundation, Rio de Janeiro, RJ, Brazil; Instituto Butantan, BRAZIL

## Abstract

The pathology of schistosomiasis mansoni, a neglected tropical disease of great clinical and socioeconomic importance, results from the parasite eggs that become trapped in host tissues, particularly in the liver and intestines. Continuous antigenic stimulation from these eggs leads to recruitment of inflammatory cells to the sites of infection with formation of periovular granulomas. These complex structures have variable size and composition and are the most striking histopathological feature of schistosomiasis mansoni. However, evaluation of granulomas by conventional microscopy methods is time-consuming and limited, especially in large-scale studies. Here, we used high resolution Whole Slide Imaging (WSI), which allows fast scanning of entire histological slides, and multiple morphometric evaluations, to assess the granulomatous response elicited in target organs (liver, small and large intestines) of two models of schistosomiasis mansoni. One of the advantages of WSI, also termed virtual microscopy, is that it generates images that simultaneously offer high resolution and a wide field of observation. By using a model of natural (*Nectomys squamipes*, a wild reservoir captured from endemic areas in Brazil) and experimental (Swiss mouse) infection with *Schistosoma mansoni*, we provided the first detailed WSI characterization of granulomas and other pathological aspects. WSI and quantitative analyses enabled a fast and reliable assessment of the number, evolutional types, frequency and areas of granulomas and inflammatory infiltrates and revealed that target organs are differentially impacted by inflammatory responses in the natural and experimental infections. Remarkably, high-resolution analysis of individual eosinophils, key cells elicited by this helminthic infection, showed a great difference in eosinophil numbers between the two infections. Moreover, features such as the intestinal egg path and confluent granulomas were uncovered. Thus, WSI may be a suitable tool for detailed and precise histological analysis of granulomas and other pathological aspects for clinical and research studies of schistosomiasis.

## Introduction

Schistosomiasis is a neglected tropical disease of great clinical and socioeconomic importance. In 2011, the World Health Organization listed 78 countries in which schistosomiasis is endemic, affecting at least 200 million people [1]. Human schistosomiasis is caused by trematode worms of the genus *Schistosoma* with most species, including *Schistosoma mansoni*, the only one that occurs in Brazil, affecting mainly the liver and the intestines [1].

The most striking histopathological feature of schistosomiasis mansoni is the development of granulomas, well-defined clusters of inflammatory cells embedded in a collagen-rich extracellular matrix around mature parasite eggs deposited in target organs (reviewed in [2, 3]). Granulomas protect host tissues by isolating toxins secreted by the egg and are necessary for egg translocation into the intestinal lumen and excretion in the feces [3]. Paradoxically, the granulomatous response is also responsible for the pathogenesis of the disease, causing severe inflammation, tissue eosinophilia, collagen deposition, fibrosis and portal hypertension [3, 4]. In the liver, granulomas may trigger severe fibrosis, which disrupts blood flow through this organ and cause portal hypertension and portacaval shunting, leading to potentially fatal esophageal bleeding [5, 6]. The intestines present a characteristic mucosal granulomatous response leading to pseudopolyposis, microulceration, and superficial bleeding [6]. Therefore, much of the symptomatology of schistosomiasis mansoni is associated to the egg-induced granulomatous inflammatory response and associated fibrosis [4].

Schistosomiasis-elicited granulomas have variable size and cell composition, depending on the evolutionary phases in which they are. The structural organization of the granulomas' stages, which is based on histological analyses, is thus complex and various classifications have been proposed and used to study both human and experimental infections [2, 7–11].

Visual analysis of histological slides on the microscope, associated or not with morphometric methods, has been classically used to study the granulomatous injury triggered during schistosomiasis mansoni [8, 9, 11]. These procedures are time-consuming and limited, especially in large-scale studies, in which analyses of numerous slides and organs are required [12]. Moreover, insufficient histological evaluation may lead to under- or overestimation of the inflammatory scenario.

Here, we used high resolution Whole Slide Imaging (WSI) for histopathological analyses of granulomas developed during schistosomiasis mansoni in two models of infection. WSI enables the acquisition of pathology information from glass slides and translate it into a digital form in a way comparable to a conventional microscope, but with several advantages such as evaluation of entire histological slides, easy image accessibility, portability, sharing, annotation, evaluation of multiple histological parameters and use for educational purposes (reviewed in [13, 14]). By using a model of natural (*Nectomys squamipes*, a wild reservoir captured from endemic areas in Brazil) [15] and experimental (Swiss mouse) infection with *S*. *mansoni* [16] we provided, to our knowledge, the first detailed characterization of granulomas in target organs (liver and intestines) with the use of WSI. WSI associated with quantitative analyses enabled a fast and reliable view of the number, type, frequency and areas of granulomas and inflammatory infiltrates and revealed a differential inflammatory response of target organs when the experimental and natural infections were compared.

## Materials and methods

### Study area

Adult specimens of *N*. *squamipes* were captured in the rural areas of the Municipality of Sumidouro (22° 02' 46" South and 42° 41' 21" West), located in the mountainous region of the state of Rio de Janeiro, Brazil, an endemic area of human schistosomiasis and where the presence of this rodent has often been registered [15]. Capture transects were established in Encanto and Pamparrão Localities along streams and irrigation channels, which constitute the habitat of this rodent. Tomahawk® traps measuring 40 cm x 12.7 cm x 12.7 cm were placed on the ground and baited with a mixture of peanut butter, banana, oat and bacon [17].

### Identification of adult worms in *N*. *squamipes*

Infected *N*. *squamipes* were identified by the presence of adult worms in mesenteric veins using perfusion of the portal-hepatic system [17]. For this procedure, a Brewer® perfusor, also known as Automatic Pippeting Machine (cat number 60480, model 40A, Scientific Equipment Products, MD, patent number 2,148,899) was used. Saline (1.8%) was inoculated through the right ventricle and the liquid obtained from the perfusion was filtered through a fine mesh fabric to retain the adult worms. Worms recovered from each infected animal were counted with the aid of a stereomicroscope. In addition to the presence of adult worms in the mesenteric veins, positivity was confirmed by parasite eggs found in stool tests [18].

### Experimental infection in mice

Swiss Webster mice aged 70 days were inoculated or not with a single inoculum of cercariae of *S*. *mansoni* (100 cercariae/mouse), LE strain. Cercariae were harvested from infected *Biomphalaria glabrata* snails, washed, counted, and injected subcutaneously into each mouse by an experienced technician. *S*. *mansoni* LE strain used in the experiments was originally isolated from a patient in Belo Horizonte, Brazil, and has been maintained in successive passages through *Biomphalaria glabrata* snails and hamsters (*Mesocricetus auratus*) at the Laboratory of Schistosomiasis (Department of Parasitology, UFMG, Brazil). Infected animals and respective uninfected controls from the same age were euthanized at 55 days or 120 days of infection. Infection was confirmed by findings of parasite eggs in the rodent feces at week five of infection [18].

### Collection of samples

Both naturally and experimentally infected animals and their respective uninfected controls were anesthetized and euthanized as before [19], and organ fragments were collected and processed for histopathological studies as below. Animals were euthanized by exsanguination (full bleed) under deep anesthesia by cardiac puncture. The anesthetic protocols included ketamine (100 mg /mL) combined with acepromazine (10 mg/mL) at a ratio of 9:1 (dose of 0.15 mL/100 g body weight).

### Ethics statement

This study was carried out in full accordance with all international and Brazilian accepted ethic guidelines and was approved by the Oswaldo Cruz Foundation Ethics Committee on Animal Use [CEUA-*Comissão de Ética no Uso de Animais*, under protocols CEUA: LW81/12 for *N*. *Squamipes* and CEUA: 32/2012 for Swiss mice). CEUA follows the Brazilian national guidelines recommended by CONCEA (*Conselho Nacional de Controle em Experimentação Animal*).

Animals (*N*. *Squamipes*) were captured under authorization of Chico Mendes Institute for Biodiversity and Conservation of the Brazilian Government (ICMBIO, authorization number 13373). All procedures with *N*. *squamipes* were carried out in the field in accordance with biosafety standards level three. Biosafety techniques and personal safety equipment were used during all procedures according to the Brazilian Ministry of Health recommendations [20].

Mice experimentally infected and uninfected controls were monitored daily for survival and well-being status (home cage evaluation, body condition, skin lesions, mobility and other general conditions) [21]. No animals died prior to the experimental endpoints (55 days or 120 days for acute and chronic phases, respectively).

### Tissue processing

Liver and intestine (small and large) samples from uninfected and infected *N*. *squamipes* (3 animals per group) and Swiss mice (6 animals per group) were removed and divided into approximately 5 mm^3^ fragments, which were immediately fixed in 4% paraformaldehyde in buffered phosphate, pH 7.3, 0.1 M overnight at 4° C. Next day, the specimens were transferred to a 0.1M phosphate buffer solution, pH 7.3 and kept in this solution at 4° C for further histological processing. Samples were then dehydrated, embedded in Paraplast^®^ (Sigma-Aldrich, USA) or glycolmethacrylate resin (GMA) (Leica Historesin Embedding Kit, Leica Biosystems, Heidelberg, Germany) [22] and cut at 3 μm (GMA) or 5 μm (Paraplast) thick sections using a Leica microtome RM2155. The histological approach combines optimal fixation and processing for visualization and quantification of inflammatory processes. Three sections of each organ were obtained at an interval of 300 μm between sections to ensure analysis of different granulomas. Sections were stained with hematoxylin-eosin (Sigma-Aldrich) or Gomori’s trichrome for qualitative and quantitative evaluation of granulomas and inflammatory processes.

### Slide processing and histoquantitative analyses

**Fig 1:**
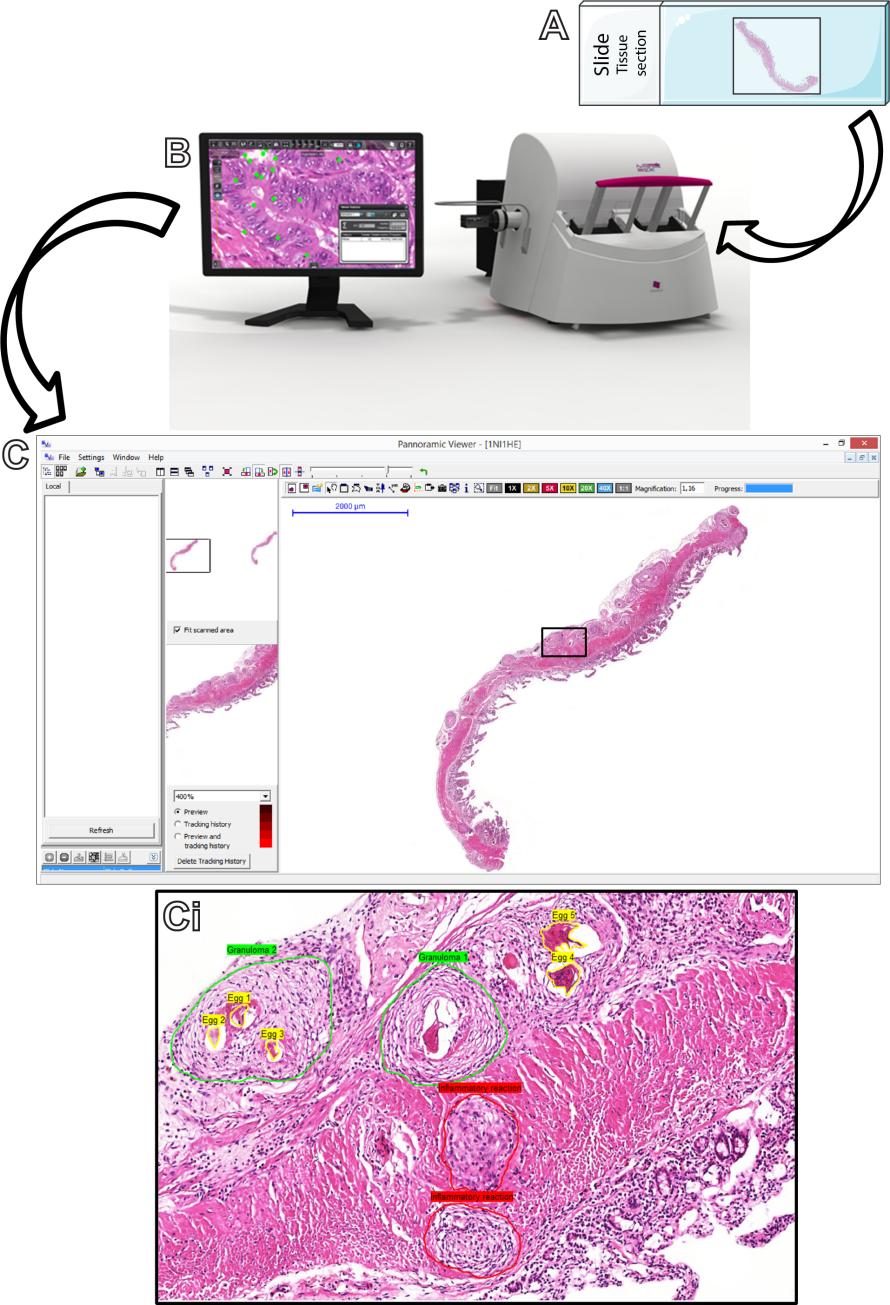
Sequence of steps to acquire and analyze whole slide images. (A) After loading the slides in the scanner, image acquisition starts with a “prescan” step in which the equipment takes a low resolution grayscale image followed by an automated setup process termed “profile” in which the tissue is detected and focused. (B) The equipment scans all regions of the slide defined by the profile and generates a virtual slide, which is saved on disk. These first steps take just few minutes and many slides can be sequentially scanned. (C, Ci) The operator can then review the virtual slide(s) and work on each one by selecting the area(s) of interest with the use of morphometric software. In the present work, the number and area of granulomas and area of inflammatory infiltrates were quantitated by using *Pannoramic Viewer 1*.*15*.*2 SP2 RTM* software. A representative digital slide shows a section from the small intestine of an *S*. *mansoni*-infected mouse in which the above parameters were manually delineated for subsequent automatic quantification.

Histological slides from the livers and intestines (small and large) were scanned using a *3D Scan Pannoramic Histech* scanner (3D Histech Kft. Budapest, Hungary) connected to a computer (Fujitsu Technology Solutions GmbH, Munich, Germany). This scanner enables a resolution of 0.23 μm per pixel. Tissue section areas (Table 1) were analyzed using *Pannoramic Viewer 1*.*15*.*2 SP2 RTM* (3D Histech kft.) or Histoquant (3D Histech kft.) softwares, which provide a morphometric detailed analysis with precise measurements of different histological parameters at high resolution of entire histological slides (Fig 1). The following morphometric parameters were evaluated and quantitated in the liver and intestine sections from the infection models: (i) Types of granulomas as described below; ii) Areas taken by granulomas: the total area related to the granulomatous response was measured as shown in Table 1; iii) Frequency of each granuloma stage; (iv) Number of eosinophils per granuloma area and proportion of eosinophils in the granulomas; (v) Area taken by inflammatory infiltrates, characterized by a large amount of accumulated leukocytes outside the granuloma. A total of 348 granulomas in *N*. *squamipes*, 202 granulomas in Swiss mice at acute phase and 444 granulomas in Swiss mice at chronic phase were recorded.

**Table 1:** Granuloma area analyzed in digital slides from target organs of *S*. *mansoni*-infected animals.

Organs	Natural infection (n = 3 animals)		Experimental infection acute phase (n = 6 animals)		Experimental infection chronic phase (n = 6 animals)	
	Total area (mm^2^)	Sections/animal	Total area (mm^2^)	Sections/ animal	Total area (mm^2^)	Sections/ animal
Liver	111.79	3	200.42	3	226.62	3
Small intestine	68.63	3	109.68	3	92.95	3
Large intestine	110.86	3	118.67	3	75.79	3

Eosinophil numbers were quantified using Histoquant software (3D Histech kft.), which enables manually marking of cells of interest and automatically counting them in the program. The numbers of eosinophils in hepatic and intestinal granulomas were quantified and results expressed in mean number of eosinophils per granuloma area in μm^2^. Additionally, the proportion of eosinophils was estimated in the most frequently found granuloma type in the livers. For this, the proportion of eosinophils was counted among 100 cells in three randomly chosen granulomas per cell section from each group (Table 1).

### Granuloma classification

The classification of evolutionary stages of granulomas was performed according to previous studies [9, 10, 23]. Thus, four main evolutionary stages of granulomas containing at least one egg of *S*. *mansoni* inside were analyzed: i) pre-granulomatous exudative (**PE**): characterized by an infiltrate of inflammatory cells in process of organization around the parasite egg; ii) necrotic-exudative (**NE**): identified by a central halo of necrosis and numerous inflammatory cells distributed irregularly on subsequent layers; exudative-productive (**EP**), characterized by a rich structure of collagen fibers and inflammatory cells concentrated in the periphery and showing a more organized and circumferential aspect; and productive (**P**), with a typical thick band of collagen fibers between the egg and few numbers of inflammatory cells.

### Statistical analysis

Two Way ANOVA followed by Tukey's post-test was used for quantitative analysis of granulomas in histological sections from different groups of infected and non-infected animals (natural and experimental infection). All analyses were performed using *Prism* 6.01 (Graphpad Software, San Diego, CA) software. The significance level was set at *P* < 0.05.

## Results

### Granuloma developmental stages in target organs of *S*. *mansoni* infection models

**Fig 2:**
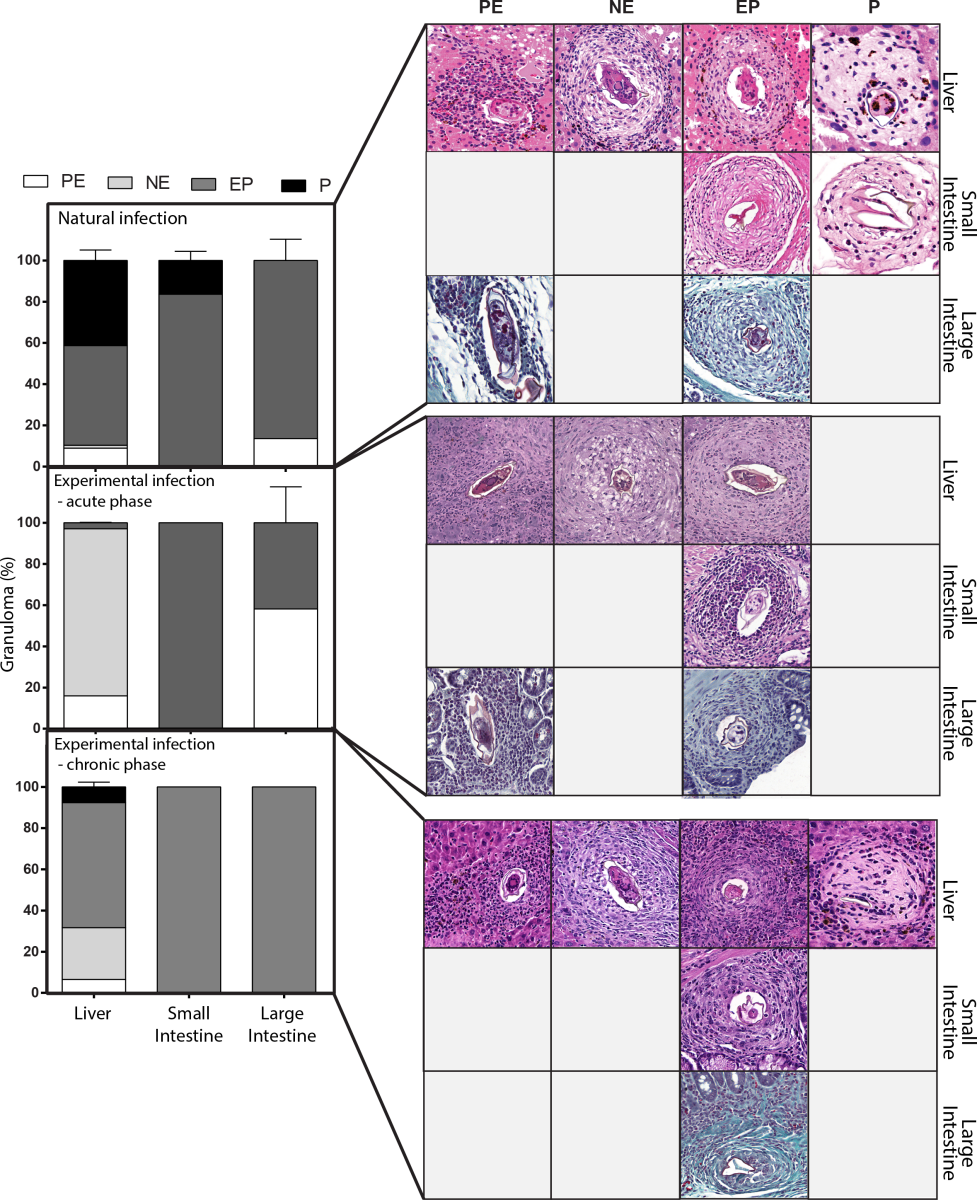
Representative types of granulomas and their frequencies in target organs of rodents naturally or experimentally infected with *S*. *mansoni*. Four types of granulomas were identified in virtual slides: Pre-granulomatous exudative (**PE**); necrotic-exudative (**NE**); exudative-productive (**EP**) and productive (**P**) as described in material and methods. Bar = 250 μm.

First, we sought to identify the occurrence and types of granulomas in the hepatic and intestinal tissues of naturally and experimentally infected animals. Our histopathological analyses using WSI showed that the granulomatous inflammatory response around the parasite eggs of *S*. *mansoni*-infected *N*. *squamipes* is a well-characterized lesion as previously documented for this rodent [23, 24]. The liver of the *S*. *mansoni*-infected *N*. *squamipes* exhibited concomitant occurrence of granulomas in different stages of maturation: PE, NE, EP and P (Fig 2). On the other hand, in the liver of Swiss mice at the acute phase of infection (55 days) only PE, NE and EP stages were observed. The P type granuloma was absent likely because this granuloma stage is observed just in the later stages of the infection [9]. Indeed, at chronic phase (120 days of infection) of the experimental infection, the four evolutionary stages of granulomas (PE, NE, PE and P) were clearly observed in infected mice (Fig 2).

Quantitative analysis of hepatic granulomas showed that naturally infected animals (*N*. *squamipes*) had a higher relative frequency of granuloma EP (48.39 ± 4.46%, mean ± SEM, *P* < 0.0001) and P (41.23 ± 2.94%, mean ± SEM, *P* < 0.0001), (Fig 2). In Swiss mice at acute phase, frequency analysis indicated a greater proportion of granulomas NE (81.16 ± 4.92%, mean ± SEM, *P* < 0.0001) while in Swiss mice at chronic stage most granulomas were EP (60.74 ± 2.33%, mean ± SEM, *P* < 0.0001) (Fig 2).

The small intestines of animals infected with *S*. *mansoni* showed granulomas EP (83.65 ± 2.58%, mean ± SE) and P (16.35 ± 2.58%, mean ± SE) for *N*. *squamipes* and only granulomas EP (100%) for Swiss mice (acute and chronic phases) (Fig 2). In the large intestines of infected animals, we found the following evolutionary stages of granulomas: EP and PE stages in both *N*. *squamipes* and Swiss mice at acute phase and EP in Swiss mice at chronic phase. Fig 2 shows the granuloma types and their respective frequencies in all three groups studied. Comparisons between each granuloma type from different organs in natural and experimental infections are shown in S1 Table.

### Differential inflammatory response elicited by natural and experimental *S*. *mansoni* infections

**Fig 3:**
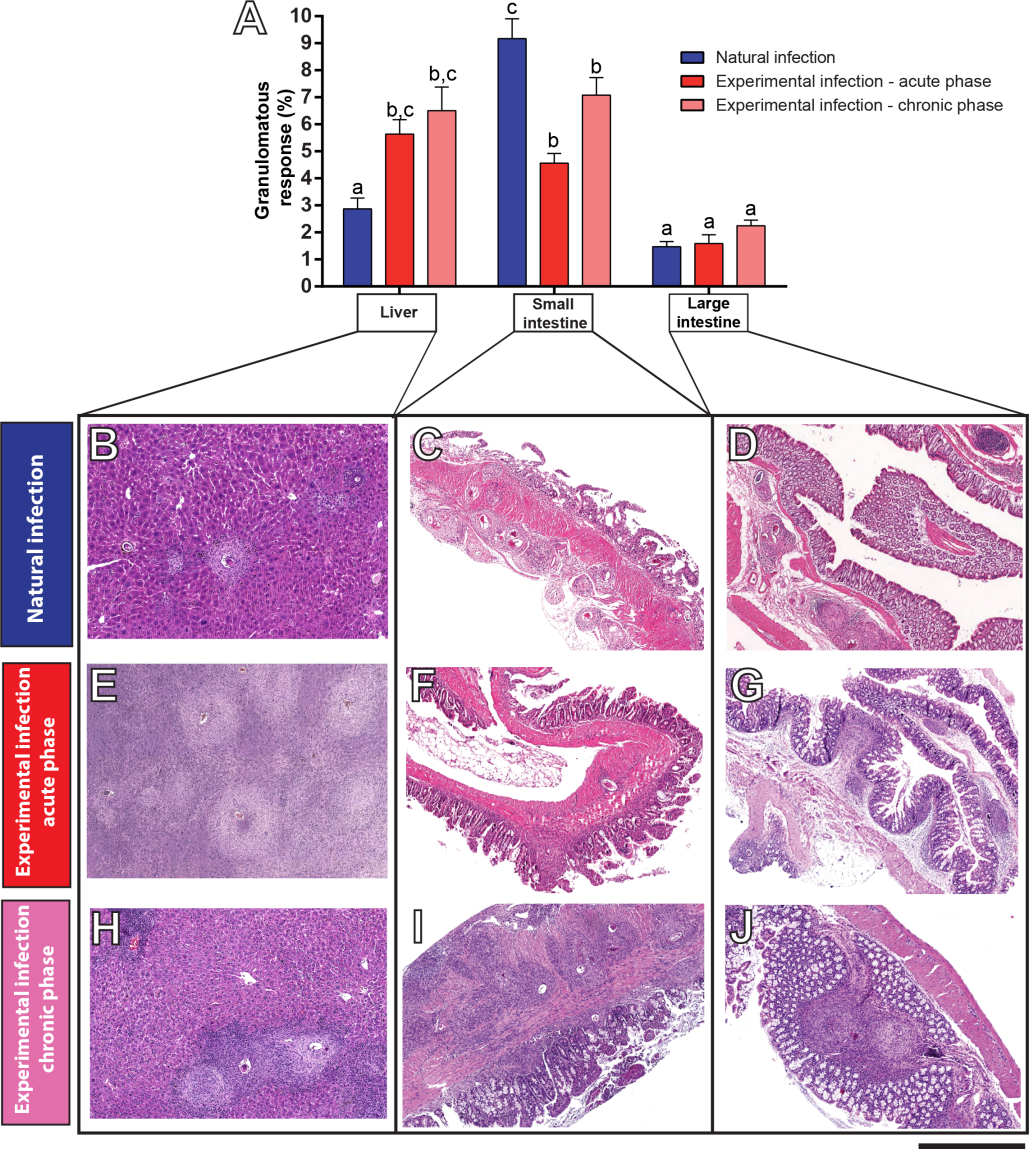
Intensity of the granulomatous response in target organs of rodents naturally or experimentally infected with *S*. *mansoni*. (A) Mean percentage of granulomatous response in livers and intestines (mean ± SEM). (B-J) Representative virtual slides of tissues with different levels of granuloma formation. In the liver (B, E, H), the lowest response is shown by the natural infection in *N*. *squamipes* (B) compared to the experimental infection in mice (E, H), while in the small intestines (C, F, I), the highest granuloma formation was observed in this wild reservoir (C). Different letters indicate significant differences between the means (*P* < 0.0001 for all comparisons between different letters in the respective groups). Bar = 1000 μm (B, D, F); 950 μm (H); 750 μm (C); 650 μm (E, G, I, J).

Having characterized the stages of granulomas, we next investigated the intensity of this inflammatory response in target organs of both models of infection. Two morphometric analyses were performed in digital slides. First, the tissue area taken by granulomas in each organ was measured and the percentage of this area in relation to the entire tissue was obtained (Fig 3). The livers from naturally infected *N*. *squamipes* showed a smaller tissue area occupied by granulomas compared to the same organ in experimentally infected mice (2.86 ± 1.20% versus 5.49 ± 1.61% and 6.50 ± 1.62% for natural, experimental acute and experimental chronic infections, respectively, mean ± SEM, *P* <0.001, Fig 3A). In contrast, when the small intestines were evaluated, we found the highest percentage of granuloma formation in the wild reservoir (9.17 ± 2.19% versus 4.67 ± 1.17% and 7.17 ± 1.05% for natural, experimental acute and experimental chronic infections, respectively, mean ± SEM, *P* <0.001, Fig 3A). In the large intestine of both models, the degree of granulomatous reaction did not significantly change when the three groups were compared (*N*. *squamipes* = 1.47 ± 0.58; mice in the acute infection = 1.59 ± 0.97; mice in the chronic infection = 2.26 ± 0.61; mean ± SEM, Fig 3). The histological structure of control, uninfected tissues is shown in S1 Fig

**Fig 4:**
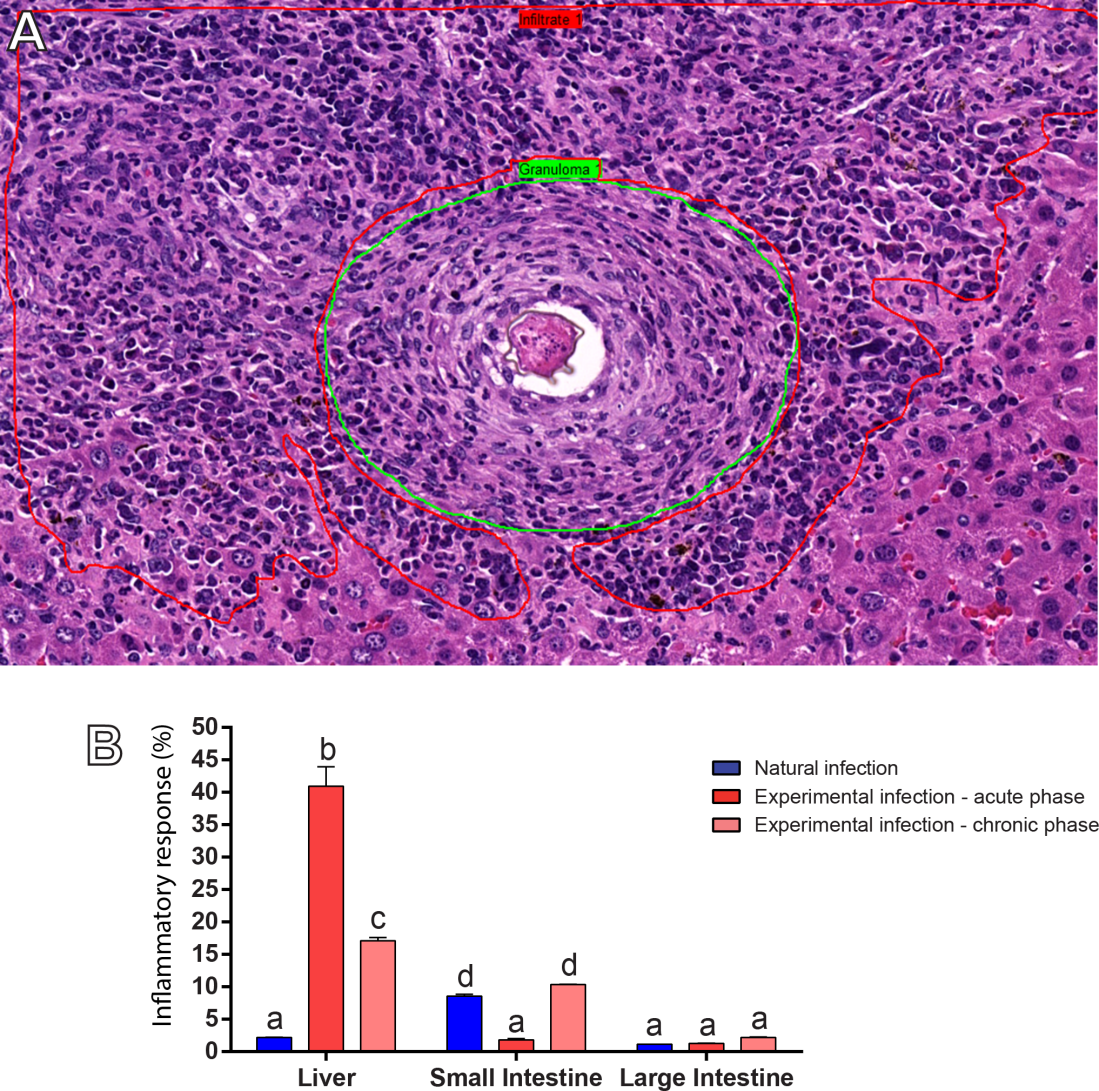
Liver and intestine areas taken by inflammatory infiltrates in the natural and experimental infections with *S*. *mansoni*. (A) Representative image of hepatic tissue from a mouse experimentally infected. After acquisition of whole slide images, areas with inflammatory infiltrates (marked in red) outside typical granulomas (marked in green) were measured. In (B), morphometric analyses reveal a very low incidence of infiltrates in the liver of infected wild rodent (natural infection) while mice experimentally infected show a very high proportion of infiltrates. In the small intestine, infiltrates are moderately higher in the natural compared to the acute experimental infection. Different letters indicate significant differences between the means (*P* < 0.0001 for all comparisons between different letters in the respective groups).

Second, the area taken by inflammatory infiltrates (tissue area occupied by leukocytes accumulated outside the granuloma) were measured (Fig 4A, marked in red). While infiltrate areas reached just 2.18 ± 0.17% of the liver in the natural infection of *N*. *squamipes*, these areas extended into 40.94 ± 9.00% and 17.12 ± 1.40% of the organ in the acute and chronic experimental infections in mice, respectively (Fig 4B, *P* < 0.0001). In the small intestines, however, inflammatory infiltrates were 8.55 ± 0.90% in the natural infection (compared to 1.80 ± 0.60% in the acute experimental infection(*P* < 0.0001), but did not differ from those found at the chronic experimental infection (*P* < 0.43). Morphometric analyses of the large intestine did not detect any statistical difference when the infiltrates were compared among the three groups (Fig 4B, *P* > 0.05).

Taken together, these results demonstrated that target organs of *S*. *mansoni* parasite are differentially impacted by inflammatory responses in the natural and the experimental infection.

### Eosinophil numbers greatly differ in granulomas from natural and experimental *S*. *mansoni* infections

**Fig 5:**
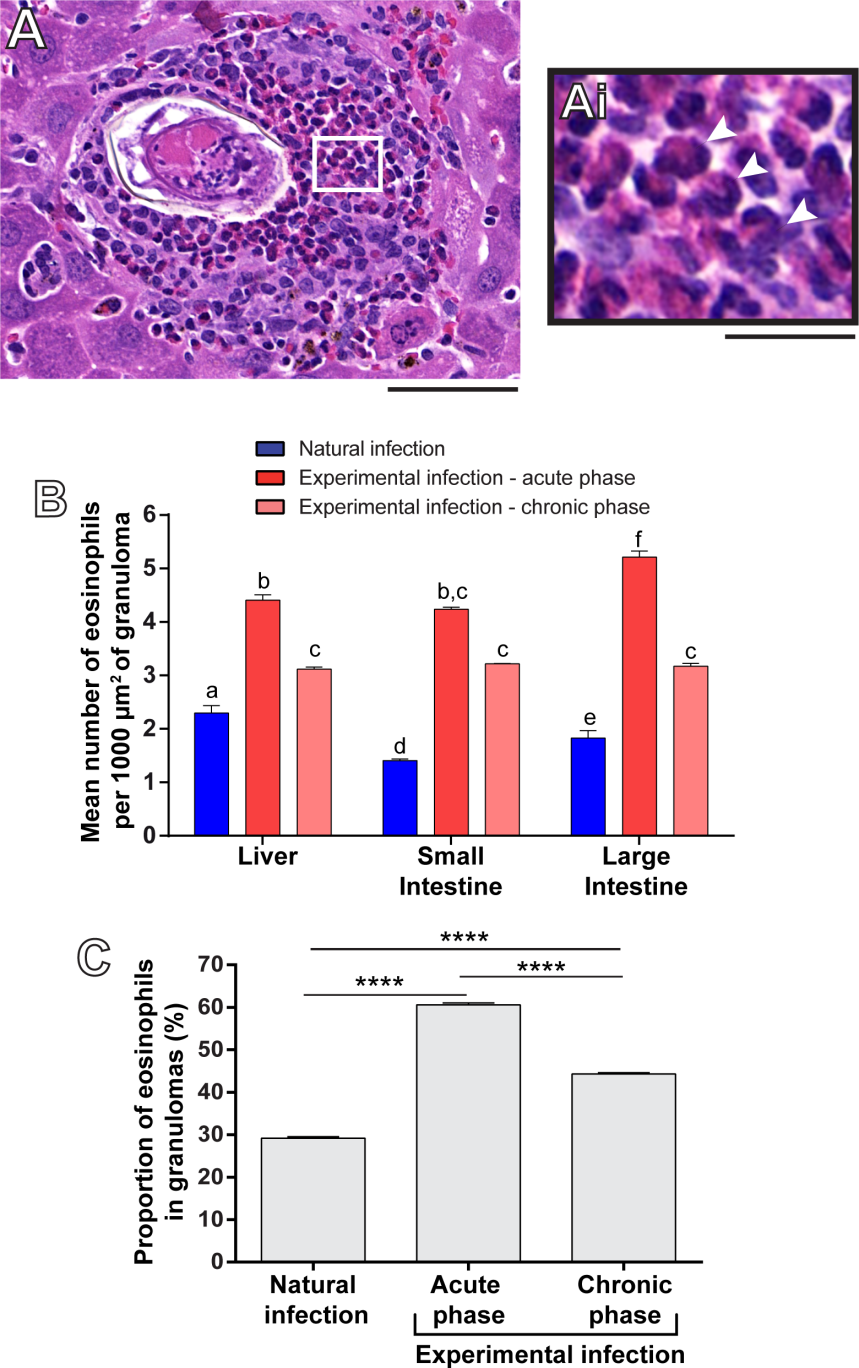
Eosinophil numbers in the natural and experimental infections with *S*. *mansoni*. (A) Representative image of a hepatic granuloma from a naturally infected wild reservoir (*N*. *squamipes*) showing accumulation of eosinophils (indicated in high magnification in Ai by arrowheads). (B) Quantitative analyses revealed a lower number of eosinophils per granuloma area (μm^2^) in all target organs in the natural infection compared to the experimental infections. Different letters indicate significant differences between the means (*P* < 0.0001 for all comparisons between different letters in the respective groups). (C) Proportion of eosinophils within hepatic granulomas in each model of the infection. (****) *P* < 0.0001. Eosinophil numbers were quantitated per granuloma area, considering all types of granuloma (B) or per most frequent type of hepatic granuloma (C). Data represent mean ± SEM. Morphometric evaluation was done with the use of *Histoquant* software. Bar = 400 μm (A), 100 μm (Ai).

The granulomatous inflammation triggered by *S*. *mansoni* is characterized by accumulation of immune cells, mainly eosinophils [3, 25, 26]. Because we found a remarkable difference in the intensity of the inflammatory granulomatous response when we compared the two models of *S*. *mansoni* infections (Figs 3 and 4), we next wondered whether the number of eosinophils within these granulomas would differ in these models. A total of 15,785 eosinophils in *N*. *squamipes*, 37,611 eosinophils in Swiss mice at acute phase and 38,540 eosinophils in Swiss mice at chronic phase was counted. By applying two morphometric evaluations in entire sections, that is, determination of the eosinophil numbers per granuloma area and proportion of eosinophils per granuloma, we demonstrated, for the first time, that the natural infection had a significant lower infiltration of eosinophils compared to both acute and chronic experimental infection in mice (Fig 5A–5C). This low number of eosinophils in the natural infection was detected in all target organs analyzed (liver, small and large intestines) (Fig 5B). Remarkably, when the proportion of eosinophils was evaluated in the most frequent type of hepatic granuloma (Fig 5C), we found that these cells corresponded to 29.20 ± 0.32% of all granuloma cells in the natural infection while in the experimental infection these numbers were 60.60 ± 0.47% and 44.30 ± 0.23% in the acute and chronic experimental infections, respectively.

### Confluent granulomas are less frequent in livers of naturally than experimentally infected animals

**Fig 6:**
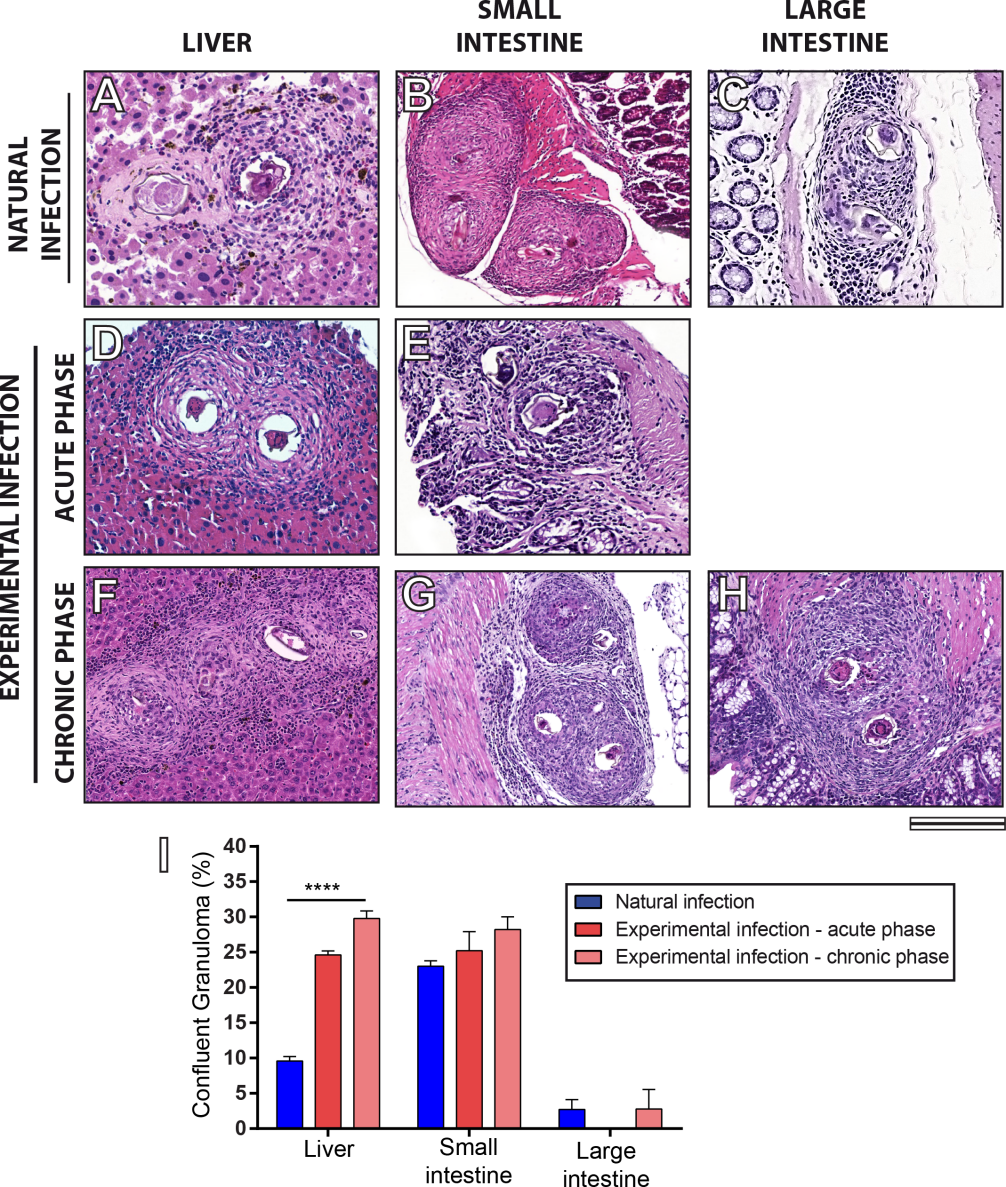
Confluent granulomas in livers and small and large intestines of *Nectomys squamipes* and Swiss mice infected with *Schistosoma mansoni*. (A-H) General morphology of confluent granulomas in different organs. Graph I shows the proportions of confluent granulomas in livers and in intestines taken from naturally infected *N*. *squamipes* (at day one of capture after confirmation of the infection) and from experimentally infected Swiss mice at days 55 (acute phase) and 120 (chronic phase) of infection. Confluent granulomas were quantified using Pannoramic Viewer software. (***) indicates significant differences between the means (*P* < 0.001). Bar = 100 μm (A, D), 220 μm (B, C); 150 μm (E, F, G, H).

To get more insights into the differential response of the natural and experimental infections with *S*. *mansoni*, we next investigated the occurrence of confluent granulomas in target organs of the disease. These granulomas, which can show multiple eggs inside, were observed in all tissues examined (Fig 6A), except in large intestines of mice at the acute phase of infection. Quantitative analyses revealed that *N*. *squamipes* had a significant lower proportion of confluent granulomas in the liver than those observed in other experimental groups (*N*. *squamipes* = 9.57 ± 0.65%; mice at acute phase = 24.60 ± 0.58%; mice at chronic phase = 29.80 ± 1.06%, mean ± SEM, *P* < 0.05). Regarding the intestines (small intestines from the three groups and large intestines from natural and experimental chronically infected groups) showed confluent granuloma ratios statistically similar (Fig 6D).

### Detection of the schistosoma egg path within the intestinal mucosa using WSI

**Fig 7:**
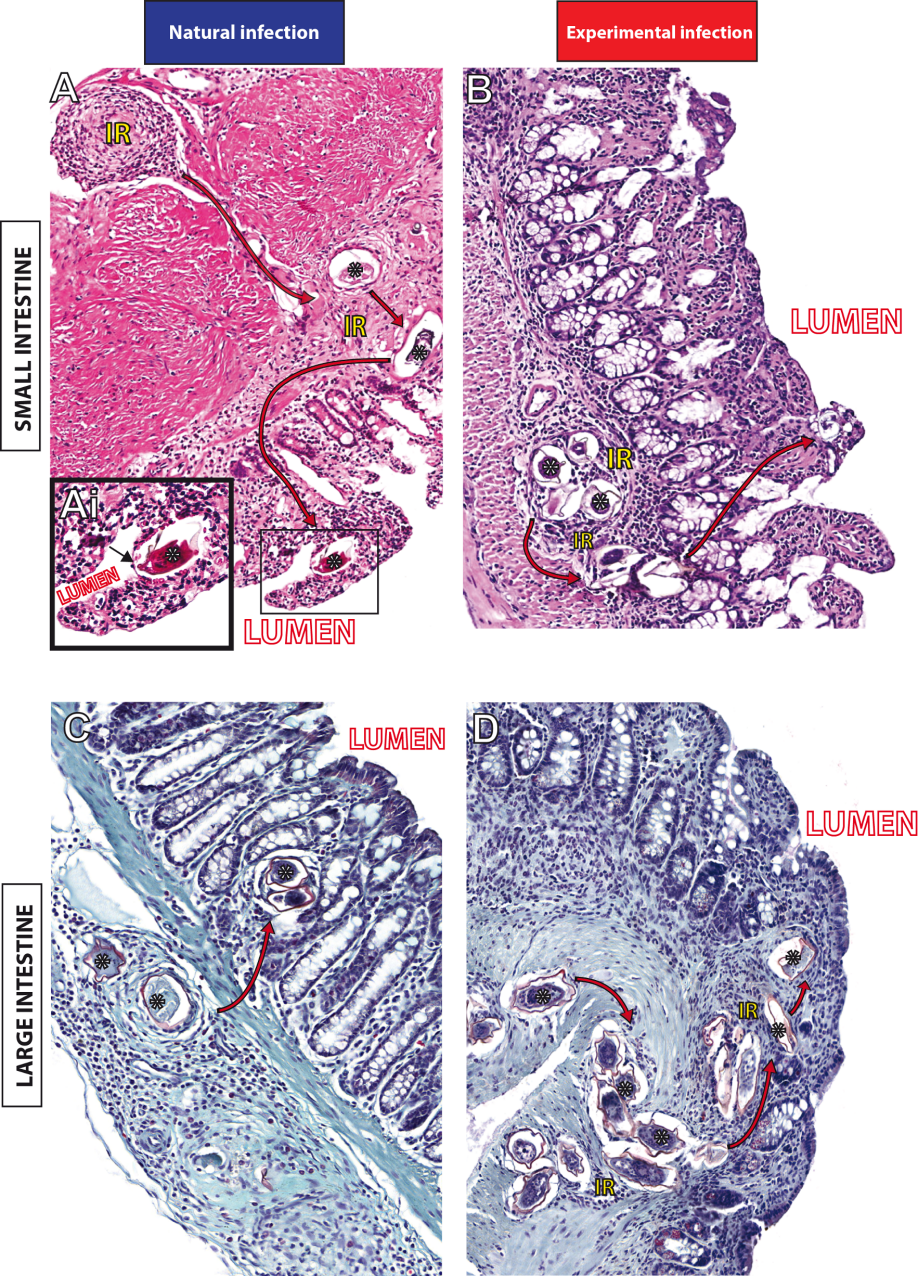
**Path of *Schistosoma mansoni* eggs in the small (A, B) and large intestines (C, D) in the rodents *Nectomys squamipes* (A, C) and Swiss mice (B, D).** The granulomatous inflammatory reaction (IR) produces a path which leads the translocation of parasite eggs (*) from the inner layers to the lumen of the intestines, as shown by the arrows. In (Ai), a *S*. *mansoni* egg is going out of the villi into the lumen of the small intestine. Liver and intestine fragments were taken from naturally infected *N*. *squamipes* (at day one of capture after confirmation of the infection) and from experimentally infected Swiss mice at days 55 (acute phase) and 120 (chronic phase) of infection. Bar = 160 μm (A); 120 μm (B, C, D); 80 μm (Ai).

In small and large intestines of animals infected with *S*. *mansoni*, granulomas act as facilitators of egg translocation into the organ layers toward the lumen to be eliminated with the feces, completing the life cycle of the parasite [3]. As noted, one advantage of WSI is the possibility to analyze large areas of tissues providing a complete panorama of pathological aspects. Thus, in parallel to the morphometric analyses of the granulomas and infiltrates, we used digital slides obtained from entire scanned sections to investigate the schistosoma egg path in the intestines of naturally and experimentally infected animals. In both small and large intestines of *N*. *squamipes* (Fig 7A and 7C) and Swiss mice (Fig 7B and 7D), the path of the schistosoma eggs within the intestinal mucosa to the lumen of the organ was clearly detected. These data confirm the fact that different hosts use this path to eliminate a large number of parasite eggs to the external environment.

## Discussion and conclusions

In pathological studies of schistosomiasis mansoni, assessment of granulomas by histological methods allows not only diagnosing the disease but also provides the basis for understanding the immunopathology of this infection [3, 11, 27–29]. Here, we applied WSI to study in detail the granulomatous response elicited in target organs (liver, small and large intestines) of two models of schistosomiasis mansoni. The use of experimental models of *S*. *mansoni* infections in mammals, especially mouse models, has facilitated our understanding on the immunopathology and pathogenesis of this disease (reviewed in [3, 30]). Thus, we chose a wild-type murine model of infection (Swiss mouse) [16, 27] for histopathological WSI application. On the other hand, natural models of *S*. *mansoni* infection have been much less studied. The water-rat *Nectomys squamipes* was chosen for our histopathological studies because it is considered one of the most important non-human hosts in the *S*. *mansoni* transmission in Brazil [15, 31–33]. One intriguing aspect of this helminthiasis in *N*. *squamipes* is that this wild reservoir is highly susceptible to this infection, but presents a notable physiological adaptation to the parasite [19, 24].

Whole slide scanners are devices that enable scanning and imaging of entire histological slides. The resulting digital images have high resolution and offer access to all areas on the slide. WSI has increasingly been used in different pathological studies to validate image-based diagnostics and for research applications [12, 34, 35]. Our data showed that WSI is a reliable tool for evaluation of the distribution, size and evolutional stages of granulomas in large areas of target organs. Our quantitative data revealed a prevalence of granulomas with necrotic-exudative features (~80%) in the liver during the acute phase of schistosomiasis mansoni in mice (Fig 2). This indicates exacerbation of inflammatory response at this stage of infection, since this type of granuloma is bulky and greatly enriched in inflammatory cells and it is also predominantly found during the acute schistosomiasis mansoni in humans [36]. As the disease evolves to the chronic phase, as a consequence of the immunomodulation, granulomas tend to reduce their sizes and become more fibrotic [11]. In fact, in the liver of chronically infected mice, we found predominance of EP and P granulomas, which are in late evolutional phases, characterized by the presence of collagen fibers and lower number of cells around the egg (Fig 2). However, newly formed granulomas (PE and NE types) were also detected since new eggs continue to reach the liver with the ongoing infection. In *N*. *squamipes* (Fig 2), WSI revealed granulomas similar in type and frequency to those found in the chronic phase of Swiss mice experimentally infected. This may be due to the multiple reinfections that the wild rodent undergoes during its lifetime with subsequent acquired immunity and/or its ability to deal with the infection.

In the small and large intestines of both infected models, WSI showed granulomas with few variations in size and evolutionary phases, with predominance of EP type (Fig 2). Other authors also found similar results when analyzing intestinal granulomas in naturally and experimentally infected rodents [37]. Thus, our data support and extend previous studies confirming that intestinal granulomas undergo no pronounced immunomodulation as that observed in hepatic granulomas [37, 38].

One of the advantages of WSI is that it generates images that simultaneously provide high resolution and a wide field of observation that can cover the entire section, extending any single field of view [12]. This aspect is critical to provide an accurate examination of the inflammatory process in its totality, which, in turn, may be used as a parameter to understand the severity of schistosomiasis with important clinical implications. Here, we evaluated the tissue area occupied by granulomas and non-granulomatous inflammation in the two models of infection. In the liver, both inflammatory responses (Figs 3 and 4) were significantly less intense in the natural infection compared to the experimental infection in mice. We also found lower incidence of confluent granulomas in the liver of naturally infected rodents. Overall, these findings indicate a high adaptability of the wild reservoir *N*. *squamipes* to the parasitism by *S*. *mansoni*. Indeed, an efficient modulation of schistosomiasis-induced periovular lesions, with no functional organ harm, has been documented in the liver of this animal [19, 23]. On the other hand, WSI of extensive areas of the small intestines revealed intense granulomatous inflammatory processes in the natural infection, significantly higher compared to the acute and even to the chronic experimental infections in mice. These findings can be associated with a more effective release of parasite eggs to the intestinal lumen since this process is dependent on the peri-ovular inflammatory cells [39]. In fact, analyses of entire virtual slides showed clearly the egg path during the process of egg release from the inner layers to the organ lumen (Fig 7). This path was shown by both infection models, but the host naturally infected with *S*. *mansoni* seems much more successful than that experimentally infected in terms of its capacity to expel the eggs and to deal with the infection [32]. As noted, the natural infection in *N*. *squamipes* “preserves” the liver and directs a more robust granulomatous response to the small intestines. In the experimental acute infection in mice, an intense inflammatory granulomatous response is detected in both organs, resulting in egg expelling but also in severe liver injury likewise observed in the human schistosomiasis [5, 6].

In addition to revealing the overall tissue morphology, WSI has the advantage to depict details of individual cells in high resolution [12]. For example, WSI can be applied to estimate mitotic activity index in cancer specimens [40]. Here, we focused on the WSI of eosinophils, cells highly elevated in number during helminthic infections, including human and experimental schistosomiasis mansoni [41]. WSI allowed an excellent detection and scoring of eosinophils within granulomas. Obviously, the quality of digital slides depends on the quality of the original slide. In the present study, we used optimal fixation and processing in combination with WSI, enabling ideal visualization and quantification of infiltrating eosinophils. Interestingly, evaluation of eosinophil numbers per granuloma area (Fig 5B) and proportion of eosinophils per granuloma (Fig 5C) showed that eosinophil infiltration is significantly lower in the natural infection in all target organs studied (liver, small and large intestines) compared to both acute and chronic experimental infections in mice.

Accumulation of eosinophils during schistosomiasis mansoni has historically been associated with a beneficial role in host defense (reviewed in [29, 41]). Eosinophils were also considered important cells during the process of *S*. *mansoni* egg release by disintegrating the epithelial basal membrane thus favoring passive expelling by intestinal peristalsis [39]. On the other hand, the presence of marked eosinophilia during this helminthiasis was also associated with a damaging inflammatory response [42].

More recently, several studies have changed the view of eosinophils as cytotoxic effector cells towards an immune-regulatory role in both adaptive and innate immunity to parasite infections, including schistosomiasis mansoni [26, 43]. Therefore, the function of eosinophils in this pathology still remains unclear and controversial. Our present findings on eosinophils revealed by WSI corroborates that the naturally infected *N*. *squamipes* is a useful alternative model for better understanding of eosinophil functions in schistosomiasis. If lower eosinophil recruitment is influencing or limiting the severity of the disease, it remains to be established.

In conclusion, a comprehensive morphological characterization of *S*. *mansoni*-induced granulomas in target organs (liver, small and large intestines) of naturally and experimentally *S*. *mansoni*-infected models can consistently be done using high resolution WSI. This approach has proved to be powerful in providing an accurate and whole view of the inflammatory response and other pathological aspects associated with the schistosomiasis mansoni.

## Supporting information

S1 Table Comparison between granuloma types in target organs from natural (*Nectomys squamipes*) and experimental (Swiss mice—acute and chronic phase) infections with *Schistosoma mansoni*. (PDF)

S1 Fig Representative histological sections of uninfected organs from *Nectomys squamipes* and Swiss mice. Note the general morphology and preserved architecture of the liver (A, B), small intestines (C, D) and large intestines (E, F) of *N*. *squamipes* (A, C, E) and Swiss mouse (B, D, F). Bar = 100 μm (A, B); 150 μm (C, F); 200 μm (D); 120 μm (E). Liver and intestine fragments were taken from naturally infected *N*. *squamipes* (at day one of capture) and from Swiss mice at days 55 and 120 of age. Histological sections were stained with hematoxylin-eosin and whole slides were scanned using a *3D Scan Pannoramic Histech* scanner.
(TIF)

## Abbreviations

WSI: Whole Slide Image
