# Supplementary material for: Histological assessment of granulomas in natural and experimental Schistosoma mansoni infections using whole slide imaging
Source: PLoS One. 2017 Sep 13;12(9):e0184696. doi: 10.1371/journal.pone.0184696 (PMC5597217; doi:10.1371/journal.pone.0184696)
Supplement: S1 Table — (PDF) [file pone.0184696.s001.pdf]

S1 Table - Comparison between the frequencies of granuloma types in target organs from natural (*Nectomys squamipes*) and experimental infections (Swiss mice - acute and chronic phase) with *Schistosoma mansoni*

	Nectomys squamipes			**Swiss acute phase**			**Swiss chronic phase**		
Granuloma types	Liver (*P* value)	Small intestine (*P* value)	Large intestine (*P* value)	Liver (*P* value)	Small intestine (*P* value)	Large intestine (*P* value)	Liver (*P* value)	Small intestine (*P* value)	Large intestine (*P* value)
PE vs. NE	0.3813	> 0.9999	0.0319	< 0.0001	> 0.9999	< 0.0001	< 0.0001	> 0.9999	> 0.9999
PE vs. EP	< 0.0001	< 0.0001	< 0.0001	0.606	< 0.0001	0.4233	< 0.0001	< 0.0001	< 0.0001
PE vs. P	< 0.0001	0.0081	0.0319	0.442	> 0.9999	< 0.0001	0.9224	> 0.9999	> 0.9999
NE vs. EP	< 0.0001	< 0.0001	< 0.0001	< 0.0001	< 0.0001	0.0029	< 0.0001	< 0.0001	< 0.0001
NE vs. P	< 0.0001	0.0081	> 0.9999	< 0.0001	> 0.9999	> 0.9999	< 0.0001	> 0.9999	> 0.9999
EP vs. P	0.4211	< 0.0001	< 0.0001	0.9925	< 0.0001	0.0029	< 0.0001	< 0.0001	< 0.0001

Granuloma classification: (**PE**) Pre-granulomatous exudative; (**NE**) necrotic-exudative; (**EP**) exudative-productive; (**P**) productive. A total of 348 granulomas in *N. squamipes*, 202 granulomas in Swiss mice at acute phase and 444 granulomas in Swiss mice at chronic phase were recorded. Differences in the frequency of granuloma types were assessed by 2-Way ANOVA followed by Tukey's post-test and the *P* values are shown.
