# Supplementary figures and images for: Histological assessment of granulomas in natural and experimental Schistosoma mansoni infections using whole slide imaging
Source: PLoS One. 2017 Sep 13;12(9):e0184696. doi: 10.1371/journal.pone.0184696 (PMC5597217; doi:10.1371/journal.pone.0184696)

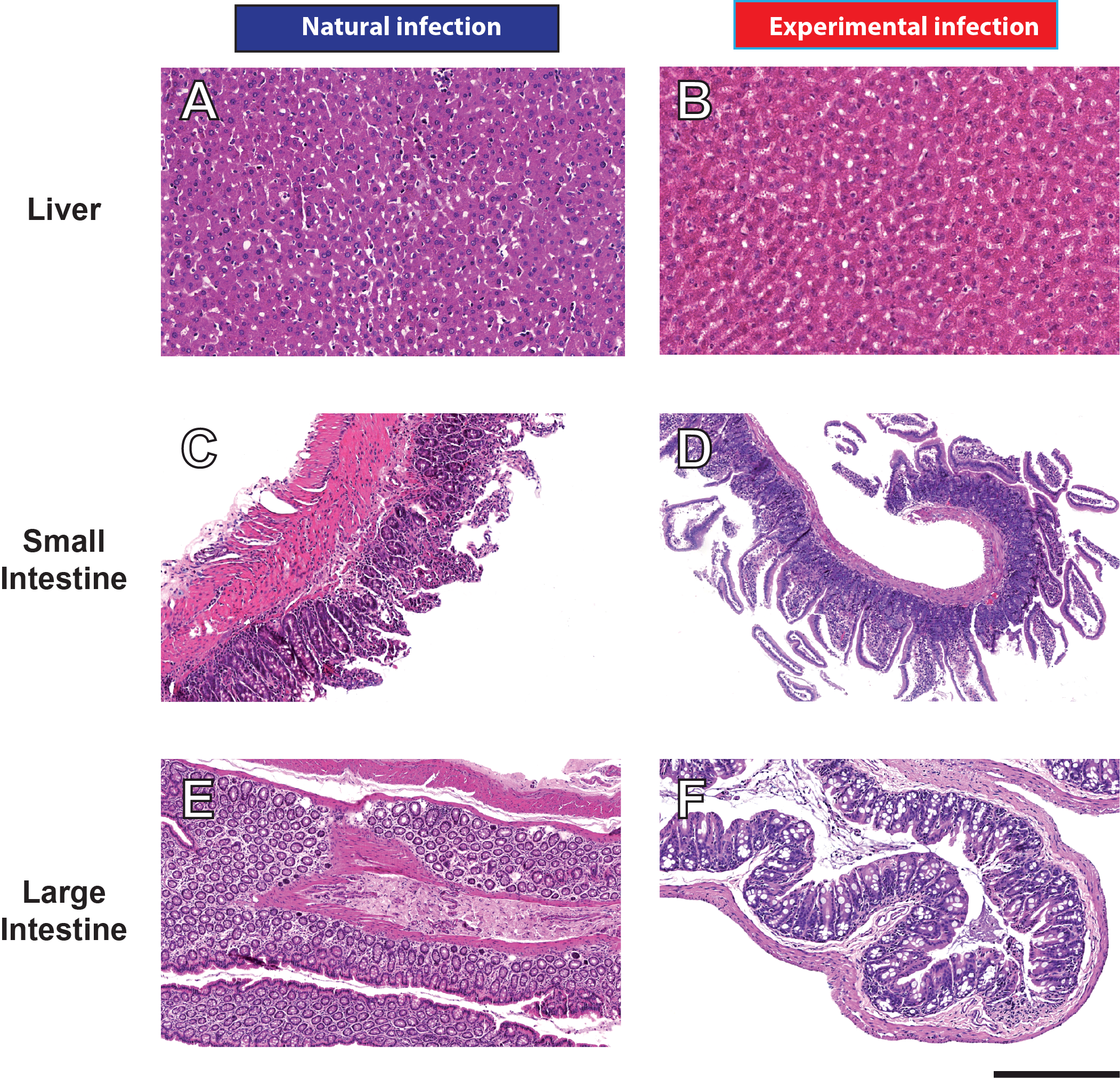

Supplement: S1 Fig — Note the general morphology and preserved architecture of the liver (A, B), small intestines (C, D) and large intestines (E, F) of N. squamipes (A, C, E) and Swiss mouse (B, D, F). Bar = 100 μm (A, B); 150 μm (C, F); 200 μm (D); 120 μm (E). Liver and intestine fragments were taken from naturally infected N. squamipes (at day one of capture) and from Swiss mice at days 55 and 120 of age. Histological sections were stained with hematoxylin-eosin and whole slides were scanned using a 3D Scan Pannoramic Histech scanner. (TIF) [file pone.0184696.s002.tif]
